# Relationship between Clinical and Immunological Features of Thyroid Autoimmunity and Ophthalmopathy during Pregnancy

**DOI:** 10.1155/2015/698470

**Published:** 2015-12-21

**Authors:** Jack R. Wall, Hooshang Lahooti, Emily J. Hibbert, Bernard Champion

**Affiliations:** Department of Medicine, Nepean Clinical School, The University of Sydney, Penrith, NSW 2751, Australia

## Abstract

*Problem*. Clinical features of Graves' hyperthyroidism (GH) generally improve during pregnancy and rebound in the postpartum period. It is unclear whether the ophthalmopathy that is associated with GH and, less often, Hashimoto's thyroiditis (HT) changes in parallel with the thyroid associated antibody reactions and clinical features or runs a different course.* Method of Study*. We retrospectively studied 19 patients with autoimmune thyroid disease over 22 pregnancies: 9 pregnancies with GH and 13 with HT. Ophthalmopathy was defined by NOSPECS class.* Results*. Thyroid peroxidase (TPO) and thyroglobulin (Tg) antibody titres decreased during pregnancy and rose in the postpartum period. During pregnancy, 5 patients with GH and 4 patients with HT developed mild ophthalmopathy and two patients with GH and HT developed new upper eyelid retraction (UER). In the postpartum period, eye scores improved in 3 patients with GH and 3 with HT, remained stable in two and 5 patients, respectively, and worsened in 2 patients with GH and one with HT.* Conclusions*. In patients with mild to moderate eye signs associated with GH and HT, the orbital and thyroid reactions ran different courses during pregnancy. Since no patient had severe ophthalmopathy, we cannot draw definitive conclusions from this preliminary study.

## 1. Introduction

It is well known that the clinical features of Graves' hyperthyroidism (GH) tend to improve during pregnancy, and smaller doses of antithyroid medication are needed to maintain euthyroidism [1], in parallel with fall in serum titres of thyroid peroxidase (TPO), thyroglobulin (Tg), and TSH receptor (TSHr) antibodies; the antibodies rebound in the postpartum period, at which time the hyperthyroidism often relapses [2]. It is presumed that Hashimoto's thyroiditis (HT) would also improve during pregnancy, but this is more difficult to assess as these patients are treated with an increased dose of L-thyroxin due to the increased needs of both mother and baby at this time.

Ophthalmopathy is associated with GH in about 50% of patients [3, 4] and mild eye and eyelid signs are found in about 25% of patients with HT [5]. The relationship between ophthalmopathy and thyroid autoimmunity is complex and poorly understood [6–8] and it is not clear whether these are components of a single disease, namely, “Graves' disease” and “Hashimoto's disease,” or separate disorders that often occur together for reasons that are unclear. A popular working hypothesis for the development of orbital inflammation in patients with thyroid autoimmunity is that immunoreactivity against a thyroid and orbital tissue shared antigen such as the TSHr or some other yet to be identified protein initiates the orbital reaction [9].

The pregnant woman with thyroid autoimmunity may serve as a good model to examine the relationship between ophthalmopathy and thyroid autoimmunity. If the eye signs were to improve during pregnancy and rebound in the postpartum period, in parallel with changes in parameters of the thyroid reaction, this would favour ophthalmopathy and thyroid autoimmunity being components of a single disease. On the other hand, if they run divergent courses, other explanations would need to be sought for their association. Here, we have studied pregnant women with GH and HT through pregnancy and in the early postpartum period, showing that clinical and immunological features of the thyroid and orbital reactions run divergent courses in about 50% of patients.

## 2. Clinical Subjects and Methods

### 2.1. Clinical Subjects

The diagnosis of GH and HT was based on standard clinical criteria and confirmed by thyroid function testing, real-time thyroid ultrasonography, and immunological tests. The demographics, baseline thyroid antibody titres, and details of any eye signs at the onset of pregnancy are summarised in Table 1. We studied 19 patients, 7 patients with GH aged 27–34 (mean age 30 yr) and 12 patients with HT aged 25–36 (mean age 29 yr) who had between them 22 pregnancies.

#### 2.1.1. Eye Assessment

The ophthalmopathy was assessed as follows: (i) a modified Clinical Activity Score (CAS) (0–12) of Mourits et al. [10] which is a measure of disease activity, (ii) Werner's NOSPECS classes [11], and (iii) upper eyelid retraction (UER) score calculated by assigning scores of 0 + (1), + (2), and ++ (3) for each of UER and upper eyelid lag, for each eye (maximum score 12). The degree of proptosis (mm) was measured using a Hertel exophthalmometer where a positive reading was defined as ≥18 mm in either eye or > 2 mm difference between the eyes. For the purpose of the study, “ophthalmopathy” was taken as a NOSPECS class ≥ 2, regardless of the CAS and significant UER, which is often the only eye sign in HT, as a UER score ≥ 2. Improvement or worsening of ophthalmopathy or UER was taken as a change in NOSPECS class of ≥1 or change in UER score of ≥1. Two of the patients with GH had ophthalmopathy and 3 had UER at the onset of pregnancy and no patient with HT had ophthalmopathy but 4 had isolated UER.

#### 2.1.2. Management of the Thyroid Disorders during Pregnancy

In patients with HT treated with thyroxin, the dose of thyroxin was usually increased by 30–50% at the beginning of pregnancy and monitored throughout pregnancy and in the postpartum period, following standard clinical practice. In patients with GH, antithyroid treatment was usually propylthiouracil during the first trimester then carbimazole (CARB) during the second and third trimesters and the dose was adjusted for maintenance of serum T4, T3, and TSH at recommended pregnancy levels. Three of the 5 patients with GH did not require any medication; CARB therapy was continued during lactation and the dose increased if there was a relapse in the postpartum period.

#### 2.1.3. Laboratory Testing

Serum free T4 (fT4), free T3 (fT3), and TSH levels were measured by Barratt & Smith Pathology using standard commercial kits according to the manufacturer's instructions. TPO and TG antibody measurements were also carried out by Barratt and Smith Pathology using standard enzyme-linked immunosorbent assay (ELISA).

#### 2.1.4. Measurement of CASQ1 and Collagen XIII Antibodies

Purified rabbit skeletal muscle CASQ1, which has 97% homology with the human protein, was supplied by Dr. Nicole Beard (ANU, Canberra, Australia) and recombinant human collagen XIII was provided by Drs. Taina Pihlajaniemi and Tu Min (Oulu University, Finland). Serum calsequestrin (CASQ1) and collagen XIII antibodies were measured in standard ELISA, as described in previous publications from this laboratory [12, 13]. Antigen concentration was 0.25 *μ*g/mL for collagen XIII and 0.5 *μ*g/mL for CASQ1, and the optimal serum dilution was 1 : 25 for both antigens. A positive test was taken as an optical density > the upper limit of normal for 30 healthy males aged < 30, namely, 194 for CASQ1 and 174 for collagen XIII.

This retrospective study was approved by the Nepean Hospital Human Ethics Committee and consent forms were not needed.

#### 2.1.5. Statistical Analysis

Statistical analysis was carried out using SigmaStat (version 2.0; Jandel Co., San Rafael, CA, USA). Prevalences of positive clinical and immunological parameters in patient groups were compared statistically using the *χ* ^2^ test or the Fisher's exact test (for five or fewer expected observations in one or more cells). Mean titres of thyroid and orbital antibodies (±SE) for patient groups at various times during and after pregnancy were compared using One Way Analysis of Variance (ANOVA) with a Bonferroni correction. A *p* value of < 0.05 was taken as significant for all analyses.

## 3. Results

We studied 19 pregnant women, 7 with GH and 12 with HT, during 22 pregnancies and in the postpartum period to assess the relationship between clinical and immunological parameters of the thyroid and orbital autoimmune reactions in this human model. The main aim was to determine whether pregnancy related changes in the orbital and thyroid reactions occur in parallel or run separate courses. The patient demographics, baseline mean thyroid antibody titres, and presence of any eye signs are summarised in Table 1.

At the onset of pregnancy, 2 patients with GH but no patient with HT had ophthalmopathy, and 3 patients with GH and 4 with HT had UER. Changes in the eye signs during pregnancy are summarised in Table 2. During pregnancy, the other 5 patients with GH and 4 patients with HT developed mild ophthalmopathy and two and one patient with GH and HT, respectively, developed new UER. In the two patients with GH and ophthalmopathy at the onset of pregnancy, eye signs improved in one and remained stable in one. UER worsened in 4 patients with GH and 4 patients with HT, remaining the same in 7 and 6 patients, respectively, and improving in two patients with GH and two with HT. Eye changes could not be assessed in one patient with GH and one patient with HT. In the postpartum period, eye changes improved in 3 patients with GH and 3 with HT, remained stable in two and 5 patients, respectively, and worsened in 2 patients with GH and one patient with HT. UER scores remained the same in 4 patients with GH and 5 patients with HT. Overall, approximately 70% of patients with thyroid autoimmunity had either improvement or no change in their eye signs while in 30% of patients the eye changes worsened, which is summarised in Table 3.

**Figure 1:**
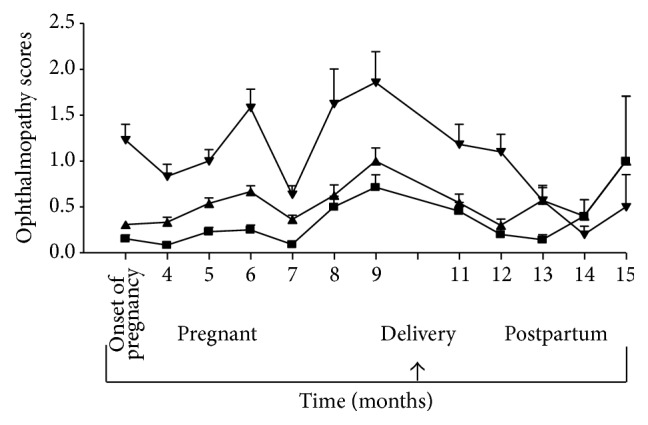
Eye changes, assessed as mean (±) Clinical Activity Score (0–12) (■), NOSPECS classes (1–7) (▲), and upper eyelid retraction score (0–12) (▼) in pregnant women with Graves hyperthyroidism and Hashimoto thyroiditis considered as a single group (thyroid autoimmunity), before, during, and following pregnancy.

Mean (±SE) CAS, NOSPECS classes, and UER scores for all patients considered as a single group (thyroid autoimmunity) before, during, and after pregnancy are shown in Figure 1. As can be seen, there were no clear patterns of change in eye signs although signs tended to worsen during pregnancy, improve near the time of delivery, and then worsen in the postpartum period, for all 3 scores (Figure 1).

Serum TPO and Tg antibodies were generally positive at the onset of pregnancy, decreased during pregnancy, in most cases to very low levels, and then increased again in the postpartum period. In the 4 patients with HT or GH who had undetectable antibody levels at the onset of pregnancy, thyroid antibodies had been positive when the initial diagnosis was made. In patients with GH, decrease in serum TPO and Tg antibody titres correlated with both a fall in TSHr antibody levels, when measured (results not shown), and improvement in hyperthyroidism, manifested by need for reducing doses of antithyroid medication. In patients with HT, it was not possible to assess any improvement or change in the thyroiditis according to clinical criteria since the thyroxin dose was routinely increased at the onset of pregnancy and monitored through pregnancy to give a TSH at the lower range of normal. It was also impossible to assess change in goitre size as a parameter of the severity of the thyroiditis.

**Figure 2:**
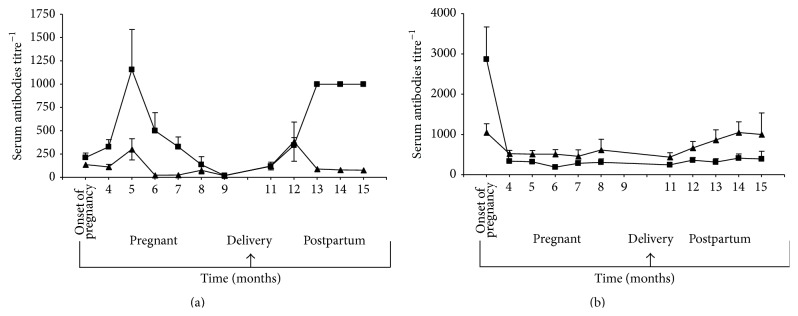
Mean (±SE) serum thyroid peroxidase (TPO) (■) and thyroglobulin (Tg) (▲) antibody titres, measured in standard enzyme-linked immunosorbent assay, in pregnant women with Graves hyperthyroidism (a) and Hashimoto thyroiditis (b) tested before, during, and following pregnancy. Results are expressed as titre^−1^.

**Figure 3:**
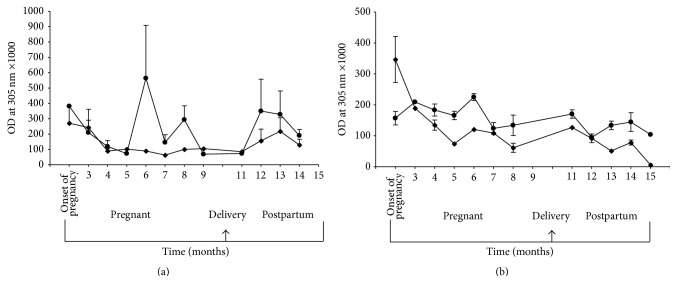
Mean (±SE) serum calsequestrin (●) and collagen XIII (◆) antibody titres, measured in standard enzyme-linked immunosorbent assay, in pregnant women with Graves hyperthyroidism (a) and Hashimoto thyroiditis (b) tested before, during, and following pregnancy. Results are expressed as optical density (OD) at 305 nm × 1000.

Mean (±SE) TPO and Tg antibody titres are shown in Figures 2(a) (GH) and 2(b) (HT), respectively; TPO levels fell quickly after the onset of pregnancy and slowly through pregnancy then rose again steeply in the postpartum period for patients with GH. There was less of a postpartum rise in TPO levels in patients with HT. However, mean (SE) titre fell to low levels during pregnancy, increased following delivery, and then fell late in postpartum period (Figures 2(a) and 2(b)). Mean (±SE) CASQ1 and collagen XIII antibody levels before, during, and after pregnancy are shown in Figures 3(a) (GH) and 3(b) (HT). Levels of both antibodies varied but tended to decrease during pregnancy and rebound after delivery, but less so than for thyroid antibodies.

## 4. Discussion

The effect of pregnancy on the course of the autoimmune reaction is complex and variable [14], although most autoimmune disorders, except for lupus and type 1 diabetes which tend to worsen at that time [14, 15], improve during pregnancy. While symptoms of GH typically improve during pregnancy and rebound in the postpartum period, there have been no studies to examine the relationship between the eye changes and thyroid autoimmunity in patients with GH and HT. In the present study, we have correlated clinical and immunological parameters of thyroid autoimmunity with those of any associated ophthalmopathy in pregnant women with GH and HT through pregnancy and in the postpartum period. To summarise the main results, while TPO and Tg antibodies consistently and markedly fell in patients with GH and HT, and while the dose of antithyroid medication decreased as fT4 and fT3 levels improved towards normal in patients with GH, overall eye changes varied considerably in pregnancy and afterwards, improving during pregnancy in 3, worsening in 2, and not changing in 2 of the patients. In patients with Hashimoto's thyroiditis, ophthalmopathy has the same general demographics, clinical characteristics, and severity and activity scores as in patients with Graves' disease. It is therefore not surprising that in our cohort the observed effects of age, gender, smoking, and TPO antibodies on the eye signs in patients with Hashimoto's thyroiditis are similar to those reported for Graves' disease [16]. In this study of UER, which is often the only feature of ophthalmopathy in HT, 3 patients improved, 4 had no change, and 3 patients worsened. Overall, approximately 70% of patients with thyroid autoimmunity had either improvement or no change in their eye signs while in 30% of patients the eye changes worsened.

Although thyroid antibodies decreased consistently during pregnancy in our patients, CASQ1 and collagen XIII antibodies, which are markers of the orbital immune reactions [7, 12–14], decreased slightly, but not markedly, over the course of pregnancy with some rebound postpartum, but this was nonsignificant and corresponding eye changes were variable. These results do suggest that the eye changes and thyroid autoimmunity do not run in parallel as one would expect if the two features were part of the same disease, that is, “Graves' disease” or “Hashimoto disease.” Thus, the development of ophthalmopathy in patients with thyroid autoimmunity may depend on the presence of additional risk factors such as smoking [17, 18] and genetic factors such as the recently identified CSQ1 polymorphism rs384216 [19].

The main limitation of the study is that no patient had severe eye disease. Ongoing studies in our laboratory of pregnant women with Graves' disease with more significant eye involvement, including eye muscle dysfunction, will provide additional information about the relationship between the thyroid autoimmune process and ophthalmopathy.

In conclusion, while these preliminary results of pregnant women with GH and HT suggest that thyroid autoimmunity and ophthalmopathy run separate courses during pregnancy, a larger number of patients with Graves' disease with more severe ophthalmopathy need to be studied before we can say that GH and ophthalmopathy, and HT and UER, are separate disorders.

## Figures and Tables

**Table 1:** Demographics, basal thyroid antibody titres, and eye signs at the beginning of pregnancy in women with Graves' hyperthyroidism and Hashimoto's thyroiditis.

Group	Age range (mean)	Eye signs at the beginning of pregnancy		Mean (± SE) thyroid antibody titre	
		OPHY^1^	UER^2^	TPO^3^	Tg^4^
Graves hyperthyroidism (*n* = 7)	27–34 (mean 30 yr)	2	3	204	138
Hashimoto thyroiditis (*n* = 12)	27–36 (mean 27)	0	4	2853	3265

^1^OPHY = ophthalmopathy.

^2^UER = upper eyelid retraction.

^3^TPO = thyroid peroxidase.

^4^Tg = thyroglobulin.

**Table 2:** Change in eye signs during pregnancy in patients with Graves' hyperthyroidism and Hashimoto's thyroiditis.

Group	Developed new ophthalmopathy		Changes in existing eye signs during pregnancy^*∗∗*^					
			OPHY^1^			UER^2^		
	OPHY	UER	No change	Better	Worse	No change	Better	Worse
Graves' disease (9 pregnancies)	5	2	1	1	5	2	1	4
Hashimoto thyroiditis (13 pregnancies)	4	1	7	0	6	6	2	4

^1^OPHY = ophthalmopathy.

^2^UER = upper eyelid retraction.

^*∗∗*^We were unable to assess changes in ophthalmopathy in one patient with Graves hyperthyroidism and one patient with Hashimoto thyroiditis.

**Table 3:** Changes in eye signs in the postpartum period in patients with Graves hyperthyroidism and Hashimoto thyroiditis.

Group	Eye changes in the postpartum period^*∗∗*^					
	Ophthalmopathy			Upper eyelid retraction		
	No change^1^	Better	Worse	No change	Better	Worse
Graves hyperthyroidism (*n* = 9 pregnancies)	2	3	2	4	0	3
Hashimoto thyroiditis (*n* = 13 pregnancies)	5	3	1	5	4	1

^1^We were unable to assess eye changes in 2 patients with Graves hyperthyroidism and 4 patients with Hashimoto thyroiditis.

^*∗∗*^We were unable to assess changes in ophthalmopathy in 2 patients with Graves hyperthyroidism and 2 patients with Hashimoto thyroiditis.
